# Locally Gated SnS_2_/hBN Thin Film Transistors with a Broadband Photoresponse

**DOI:** 10.1038/s41598-018-28765-4

**Published:** 2018-07-12

**Authors:** Dongil Chu, Sang Woo Pak, Eun Kyu Kim

**Affiliations:** Quantum-Function Research Laboratory and Department of Physics, Hanyang University, Seoul, 04763 South Korea

## Abstract

Next-generation flexible and transparent electronics demand newer materials with superior characteristics. Tin dichalcogenides, Sn(S,Se)_2_, are layered crystal materials that show promise for implementation in flexible electronics and optoelectronics. They have band gap energies that are dependent on their atomic layer number and selenium content. A variety of studies has focused in particular on tin disulfide (SnS_2_) channel transistors with conventional silicon substrates. However, the effort of interchanging the gate dielectric by utilizing high-quality hexagonal boron nitride (hBN) still remains. In this work, the hBN coupled SnS_2_ thin film transistors are demonstrated with bottom-gated device configuration. The electrical transport characteristics of the SnS_2_ channel transistor present a high current on/off ratio, reaching as high as 10^5^ and a ten-fold enhancement in subthreshold swing compared to a high-κ dielectric covered device. We also demonstrate the spectral photoresponsivity from ultraviolet to infrared in a multi-layered SnS_2_ phototransistor. The device architecture is suitable to promote diverse studied on flexible and transparent thin film transistors for further applications.

## Introduction

An emerging new two-dimensional (2D) material is metal tin dichalcogenides, which have a layered structure composed of an earth-abundant compound solid. It is currently being considered as a promising candidate for flexible and heterostructured electronics^1–3^. Remarkably, the tin-based chalcogenide alloy SnS_2−x_Se_x_ shows a broad modification of the band gap with a selenium composition (for example, 2.07 eV and 0.97 eV for SnS_2_ and SnSe_2_, respectively)^4–6^, which provides new possibilities for optoelectronics^7^. Only a few studies have investigated the mechanical characteristics of tin dichalcogenides so far. However, the covalently bonded SnS_2−x_Se_x_ alloy has shown that its lattice structure has a hexagonal CdI_2_-type, analogous to the widely investigated molybdenum disulfide (MoS_2_); this led us to expect that the alloy would have a higher strain limit than that of ionic-bonded bulk semiconductors^8^. Moreover, Mitzi *et al*. demonstrated that the soluble semiconducting SnS_2−x_Se_x_ can offer solution-processed thin films, making the integration of polymer substrate accessible^1^. All of these properties give this type of material great potential to meet the criteria for wearable and flexible devices^8^.

Pan *et al*. investigated an SnS_2−x_Se_x_ crystal-based thin film transistor (TFT) under different x-contains, finding that the current on/off ratio was heavily decreased in the selenium-rich channel because of its large electron concentration^9^. This has motivated significant efforts to investigate the SnS_2_ crystal for field-effect transistors (FETs)^10,11^ and photodetector applications^12–14^. For instance, an SnS_2_ nano-membrane FET with universal back-gated device geometry reported by De *et al*. exhibited a high switching ratio of up to 10^6^ and a poor subthreshold swing (SS)^11^. Other works have confirmed this finding and further shown that the SS parameter was typically observed in dozens of volts per decade range. The device architecture in the form of a top-gated FET capped by a high-κ Al_2_O_3_ layer demonstrated similar subthreshold swing values approximately 10 V/decade^10^. It is believed that the trapped charges located between 2D and conventional oxide significantly influence the quality of the interface. Because layered solid crystals lack dangling bonds, the materials provide primary advantages in building heterostructures that combine diverse 2D layers into a three-dimension^15^. However, no study has yet reported research of transistors encapsulated by a wide band-gap 2D dielectric (5–7 eV)^16,17^ that is a hexagonal boron nitride (hBN) with an integrated high-quality SnS_2_ nanosheet.

In this study, we construct a multi-layered SnS_2_ channel device incorporating hBN as a gate dielectric. Taking a different approach from other published works, the proposed transistors have a locally gated geometry instead of using universal silicon back-gating. We report a substantial improvement of the SS parameter of the device and characterize the effect of the Schottky-limited metal/semiconductor contact to describe the thermally activated transport. Furthermore, for the phototransistor, we also include the photoelectric behavior of the light-exposed SnS_2_. It appears that the SnS_2_ crystal responds to a wide range of photon spectra.

## Results

**Figure 1:**
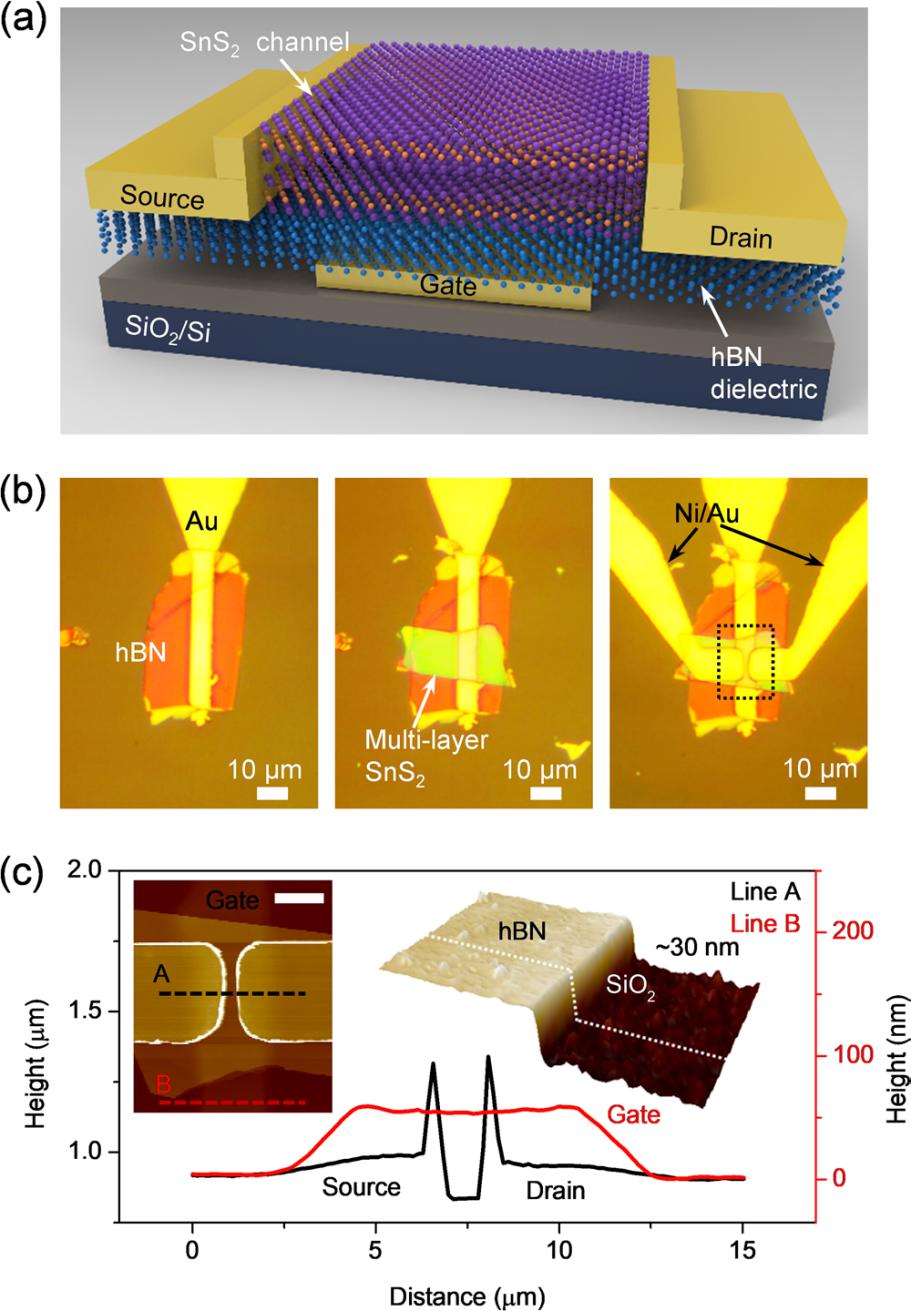
(**a**) 3D schematic representation of a bottom-gated SnS_2_/hBN heterostructure transistor. The high-quality hBN insulator proves to be an ultra-flat surface acting as a substrate for the precisely aligned SnS_2_ layer. (**b**) Optical images of the deposition of the hBN flake on the gold gate (left), the transfer of SnS_2_ (thickness typically in 1–30 nm range) onto the top of the hBN (middle), and the defined metal leads for source/drain contact (right). (**c**) The height profiles for lines A and B acquired from the AFM image (inset, left) of the device. The scale bar is 5 μm. Inset (right): 3D topography for the hBN on the SiO_2_, showing about a 30 nm thickness.

**Figure 2:**
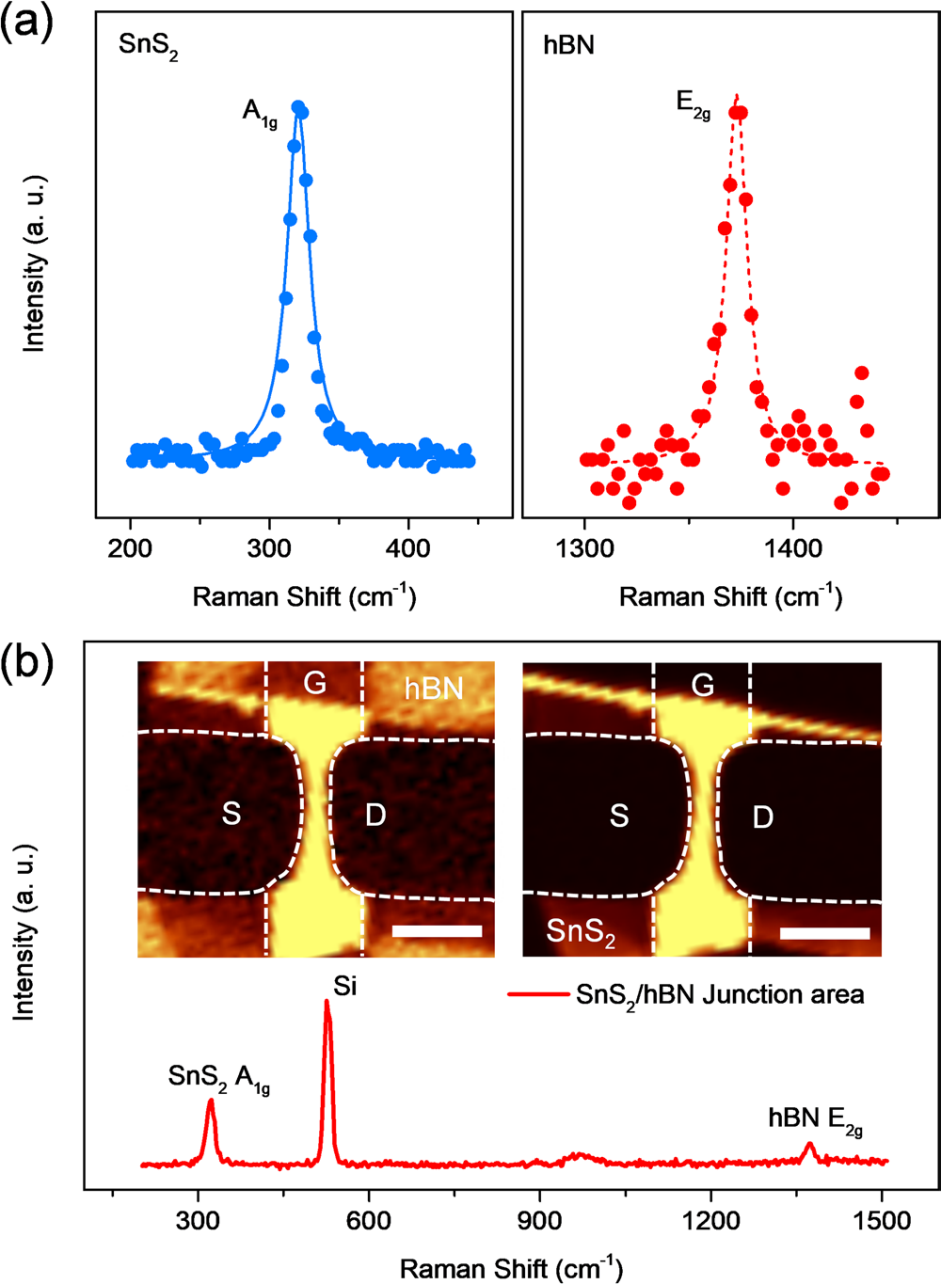
(**a**) Raman spectra of the multi-layered SnS_2_ (left panel) and the hBN (right panel). From the data, the Raman peaks for SnS_2_ and hBN occur respectively at 320.6 and 1372.4 cm^−1^. (**b**) Raman signal taken in the SnS_2_/hBN heterostructure region, showing peaks for the A_1g_ mode of SnS_2_ and E_2g_ mode of hBN. Insets: Raman frequency mapping image displaying the E_2g_ peak intensity (left) and the A_1g_ peak intensity (right), respectively, for hBN and SnS_2_. The scale bar is 5 μm.

Figure 1a schematically illustrates the device geometry of the proposed SnS_2_/hBN transistors, where atomically flat hBN acted as the gate dielectric^18^ and a multi-layered SnS_2_ nanosheet was used as the carrier transport layer. Constructing the bottom encapsulation of hBN is beneficial because the SnS_2_ layer is far from the underlying potential fluctuation (SiO_2_ substrate). Our recent investigation showed that the interface trap sites located at the 2D/SiO_2_ interface could represent more than 10^12^ states/cm^2^eV^19^. Because contamination should be avoided during the fabrication process, we used a polymer-incorporated Scotch-tape residual-free technique to minimize the use of chemical solvents. In contrast to the wet transfer method described in other reports, this technique has the ability to control the interface trap state D_IT_ down to the 10^10^ states/cm^2^eV range for a suspended 2D channel structure^19^. In this work, we fabricated more than five SnS_2_ devices with typical S/D dimensions: a channel length/width (L/W) ratio of 0.3 and a gate lead with a width of 5 μm. Optical images of the step-by-step preparation of the hetero-structured device are depicted in Fig. 1b. The as-made devices were subsequently characterized via atomic force microscope (AFM) analysis to quantify the thickness of the hBN dielectric and the SnS_2_, as illustrated in the left inset of Fig. 1c. The AFM cross-sectional profiles labeled *line A* (black) and *line B* (red) indicate a clear overlap between the channel area and the local gate, as illustrated in Fig. 1c. For the material characterization, the Raman spectra of the as-exfoliated SnS_2_ exhibit two non-degenerated scattering modes, with the out-of-plane A_1g_ mode located at 320.6 cm^−1^ and the weak in-plane E_g_ peak located at 213.5 cm^−1^ (see the Supplementary, Fig. S1) under room temperature (see Fig. 2a, left). The data are in agreement with those of previous works^20,21^. On the other hand, in-plane mode, E_2g_ of hBN is displayed in Fig. 2a right in consistent with other literature (The full Raman spectra of the heterojunction area can be seen in Fig. 2b)^22^.

**Figure 3:**
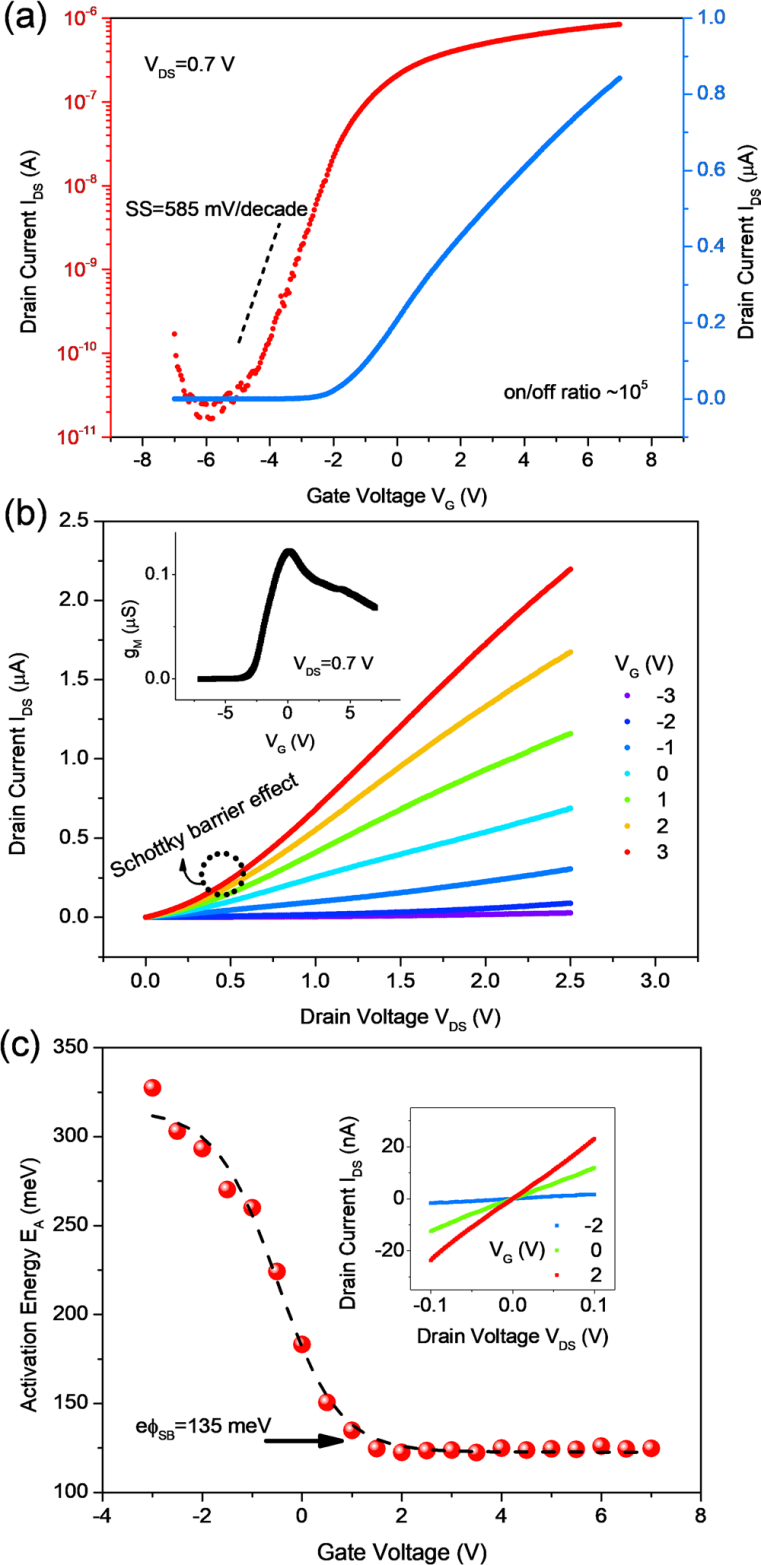
(**a**) Semi-log (left axis, red) and linear (right axis, blue) scale I_DS_–V_G_ transfer characteristics of the multi-layered SnS_2_ transistor biased at V_DS_ = 0.7 V. The device exhibited an SS as low as 585 mV/decade and an on/off ratio of approximately 10^5^ at room temperature. (**b**) I_DS_–V_DS_ output curves for various applied gate bias values from −3 to 3 V. The region in the black circle shows the nonlinear property. Inset: transconductance versus bottom-gate voltage at V_DS_ = 0.7 V, resulting in a maximum g_M_ peak of 0.12 μS. (**c**) The extracted activation energy as a function of applied gate voltage. The Schottky barrier height is evaluated to be 135 meV for the Ni/SnS_2_ interface. Inset: I-V characteristics under small V_DS_ bias.

Next, we proceed to examine the SnS_2_ channel TFTs and the influence of the hBN on the SS performance. Figure 3a shows the drain current I_DS_ behavior of the devices as a function of the gate voltage V_G_ under a constant drain voltage V_DS_ of 0.7 V, exhibiting an SS of 585 mV/decade in a 30-nm-thick hBN inserted device. The different drain voltages also revealed similar subthreshold swing slope characteristics (see Supplementary, Fig. S2a). This value is one order of magnitude smaller than that of a high-κ (Al_2_O_3_) covered SnS_2_ transistor (in a top-gated configuration)^10^. It is well known that SS = ln(10)(k_B_T/e)(1 + η) and η = (C_D_ + C_IT_)/C_BN_, where k_B_ is the Boltzmann constant, T is the absolute temperature, e is the elemental charge, C_D_ is the depletion capacitance, C_IT_ = e^2^D_IT_ is the interface trap capacitance, and C_BN_ is the bottom-gate capacitance^19^. Thus, our devices demonstrated a factor η ≈ 8. Despite implications that high-quality SnS_2_/hBN contact is indicated, the research has not yet fully explained how the interfacial quality is correlated with the electronic characteristics, especially for a gate stack. Nonetheless, we attribute such SS enhancement to the highly coupled interface with negligible chemical residues. Hysteretic effect in I_DS_-V_G_ characteristics often reflects the quality of channel/dielectric junction. The interfacial quality was further confirmed by the forward and backward direction sweeping of I_DS_ − V_G_ transfer curves which results a negligible hysteresis by amount of <200 mV as displayed in Fig. S2b. Consistent results were observed in all of the samples with current switching ratios in the 10^4^ to 10^5^ range and n-type conduction, as described in the literature^9,11,13^. The transconductance g_M_ defined by dI_DS_/dV_G_ displays a maximum value of ~0.12 μS at V_DS_ = 0.7 V, as displayed in the inset of Fig. 3b. An important figure of merit of the transistor field-effect mobility μ_FE_ is determined by the relationship μ_FE_ = g_M_/C_BN_V_DS_ × (L/W), where C_BN_ = ε_BN_ε_0_/d_BN_ (d_BN_ = 30 nm is the thickness of the hBN layer and ε_BN_ = 3–4 is the dielectric constant of the hBN)^23^. As a result, μ_FE_ is calculated to be 0.1–0.5 cm^2^/Vs, comparable to those of single-layered MoS_2_ (0.1–10 cm^2^/Vs range)^24^. It should be note that different growth method commonly influences the electrical properties of SnS_2_ crystal. Song *et al*.^10^, De *et al*.^11^ and Ahn *et al*.^33^ reported the mobility of approximately 1–2 cm^2^/Vs from SnS_2_ grown by vapor transport technique. However, the SnS_2_ solid crystal prepared by vertical Bridgman technique have showed poor mobility around 0.1 cm^2^/Vs^13^. Beside contact engineering and dielectric interface improvement, the material’s mobility seems influenced by growth method of SnS_2_.

To establish efficient carrier injection from outside (e.g., S/D metal), which is needed to enhance the device performance, the contact issues have been preliminarily investigated for the MoS_2_ system, and several novel approaches have been suggested^25^. So far, the ohmic contact formation for SnS_2_ materials is still unclear. Nevertheless, a linear increment in I_DS_ can be observed for different V_G_ bias conditions, suggesting an ohmic-like contact at the nickel/SnS_2_ junction at a small V_DS_ bias range, as depicted in the inset of Fig. 3c. However, an ambiguous result emerges when the V_DS_ is extended to a few voltages (quasi-linear region, before current saturation): a slightly nonlinear dependence of I_DS_ is found (indicated by the black circle) in I_DS_–V_DS_ output characteristics of the SnS_2_ device, as shown in Fig. 3b. We attribute this nonlinear behavior to the rise of the Schottky barrier height, eϕ_SB_ because a contact mismatch occurs between the high work function, W_F_ of nickel metal (W_F_ = 5.2 eV)^26^ and the electron affinity, eχ_s_ of SnS_2_ (eχ_s_ = 5.0 eV)^27^. Owing to the similarity in crystal structure and chalcogenide compound compared with MoS_2_ layered material, similar consequences could be expected for other 2D systems. To better address this point, we measured the temperature-dependent I–V characteristics as temperature varied from 300 to 410 K (Supplementary Information, Fig. S3). Carrier transport across a metal–semiconductor barrier involves a quantum mechanical tunneling and a thermionic-emission process, so that the devices measured at high temperature regime allowed suppression of the tunneling current contribution^25^. At a high temperature regime, an expression similar to the Arrhenius equation and also known as thermally activated transport model can be derived as g_DS_ = g_0_exp(−E_A_/k_B_T), where g_DS_ = dI_DS_/dV_DS_ is the conductance, and g_0_ is the fitting parameter^19,25^. The conductance g_DS_ fitted with this equation is depicted in Fig. S3b (see Supplementary Information). The activation energy E_A_ as a function of V_G_ acquired from Fig. S3b is illustrated in Fig. 3c. In this plot, we can determine a 135 meV of eϕ_SB_ for Ni/SnS_2_ contact by evaluation of the starting point of deviation from the linear response by following Radisavljevic and Kis^28^. Such Schottky barrier determination is based on activation energy measurement. The details of the evaluation method of ϕ_SB_ can be found in other literatures^28,29^ as well as our previous pulications^19,25^. A measured E_A_ = 0.18 eV at zero V_G_ generally indicates the position of the impurity donor level with respect to the conduction band of SnS_2_, and the activation energy is close to the value of 0.14 eV reported by Pan *et al*. and the value of 0.13 eV reported by De *et al*.^9,11^.

**Figure 4:**
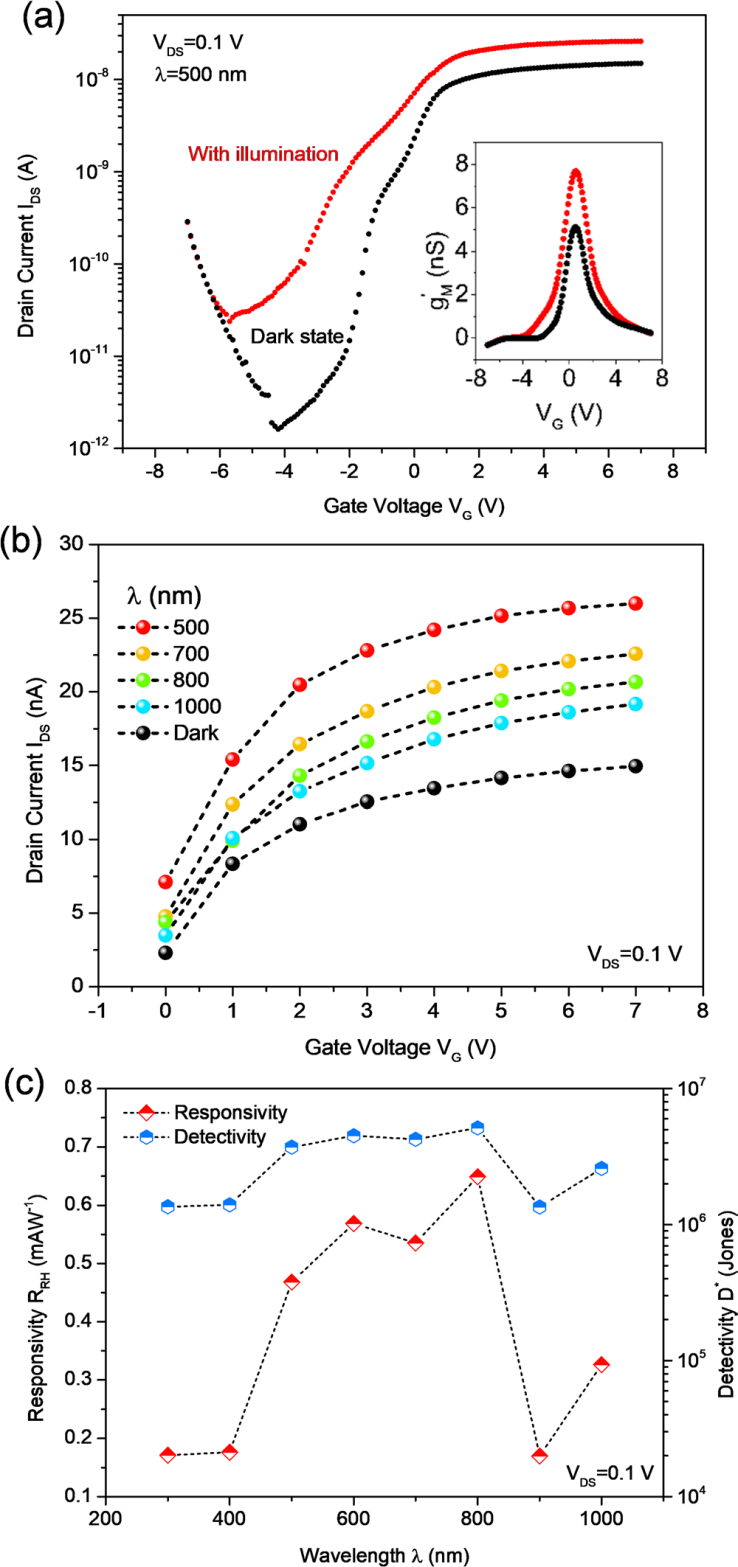
(**a**) Semi-log I_DS_–V_G_ transfer characteristics of the SnS_2_-based phototransistor for the dark state and for 500 nm wavelength illuminated curves at V_DS_ = 0.1 V. Inset: the effective transconductance of the device as a function of gate voltage. Black and red curves are the device under dark condition and 500-nm-wavelength light exposure, respectively. (**b**) Linear scale of transfer curves for different wavelengths (ranging from 500 to 1000 nm) in the accumulation region. (**c**) Photoresponsivity and detectivity of the device as functions of wavelength at V_DS_ = 0.1 V, showing a maximum R_PH_ of 0.65 mA/W.

Figure 4a shows the I–V transfer curves with (photon energy of 2.48 eV, green line) and without (dark state, black line) monochromatic light illumination at V_DS_ = 0.1 V. The current under illumination I_ILL_, defined as I_ILL_ = I_PH_ + I_DA_ (I_PH_ and I_DA_ are the photocurrent and dark current, respectively), exhibits a dramatic I_DS_ increment of the SnS_2_ phototransistor in both the on and off states of the device, whereas the incident light with an intensity of 23.5 μW has about a 30-fold influence in the off-state and 2-fold in the on-state. With light illumination on different states of the device (on-and off-states), the devices exhibit different photocurrent response. Lowering the Schottky barrier (on-state), an additional photocurrent excited by photo-induced band-to-band transition contributes to the drain current. Raising the Schottky barrier (off-state) restricts the dark current, resulting in a more pronounced photocurrent extraction. Therefore, we carefully conclude that photo-excited carrier transport primarily dominates over the thermionic and tunneling current, which is in agreement with other publications^30,31^. We found that the effective transconductance g_’ M_ of the SnS_2_ channel under light illumination showed clear increasement compared to the dark state, as depicted in the inset of Fig. 4a. The SnS_2_ phototransistor was further exposed to different monochromatic lights ranging in λ from 500 to 1000 nm, representing the series photo-induced I–V transfer properties at the on-state of the device with V_DS_ = 0.1 V, as displayed in Fig. 4b. Electron-hole pair generation by optical means usually requires an incident photon energy close to the band gap of the multi-layered SnS_2_. Interestingly, the device weakly responds to light with a long λ (such as 600 nm, corresponding to 2.07 eV), implying an extrinsic type of the phototransistor with a defect-assisted energy level introduced. This effect can be explained by the defect-level involvement of the band gap of SnS_2_; the transition between the defect-level and the conduction/valence band edge can contribute to I_PH_^29^. Photoresponsivity, R_PH_ is an important metric of the phototransistor and is estimated by I_PH_/P_L_, where P_L_ is the optical power (see Fig. S4) and the broad spectral response is shown in Fig. 4c. In this photoresponsivity calculations, calibration of device’s active area is excluded. The device performance exhibits an R_PH_ of 0.47–0.65 mA/W at the visible light range and is reduced to 0.33 mA/W at infrared due to the weak light absorption with an applied gating of 7 V. The measured R_PH_ as function of V_G_ is given in Fig. S4a. The responsivity of SnS_2_/hBN devices is lower than that of MoS_2_ based phototransistor (over 343 A/W)^30^, but it is higher than that in a SnS_2_ nanosheet photodetector reported by Tao *et al*. (around 1.13 × 10^−3^ mA/W under 532 nm photon wavelength)^32^ and vapor transport synthesized SnS_2_ crystal reported by Ahn *et al*. (within 0.1–1 mA/W range)^33^. Alternatively, optical characteristics of SnS_2_ could be highly improved by synthesis technique, such as chemical vapor deposition (CVD) for minimizing sulfur vacancy. Yang *et al*. reported that the SnS_2_ flake photodetectors prepared by CVD method archive significant improvement in photoresponsivity exceeding 1.19 A/W at 400 nm light^34^. Analogues to MoS_2_ crystal fabrication, different growth process may create different amount of sulfur vacancy in SnS_2_ which have great impact on photodetector application as discussed by Xie *et al*. (details see their publication)^35^. Therefore, we believe that extrinsic type of device with wide spectral response is probably due to sulfur vacancy induced deep states near bottom of conduction band.

Another key parameter is the detectivity, D^*^ which is the reciprocal of the noise equivalent power, given by D^*^ = R_PH_A^1/2^/(2eI_DA_)^1/2^. Here, A is the device effective area. The calculated D^*^ value showed a typical range of 1.4 × 10^6^ to 5.1 × 10^6^ Jones at V_DS_ = 0.1 V and V_G_ = 7 V. Furthermore, the external quantum efficiency, EQE is measure of the ratio of the number of carriers produced by the number of photons. The EQE can be converted from R_PH_ by employing EQE = R_PH_hc/λe, here h and c are Plank constant and speed of light, respectively. We observed approximately 0.1% of EQE at visible light range.

## Discussion

We fabricated SnS_2_/hBN heterostructured devices and characterized the devices by electrical and optical measurements techniques. The interfacial behavior between the SnS_2_ and hBN layered crystal is discussed. The locally placed gate separated by an hBN insulating layer presented an efficient modulation of the channel conductance with a current on/off ratio of up to ~10^5^. The insertion of an ultra-flat dielectric layer allowed the device to exhibit SS values as low as 585 mV/decade. The detailed temperature-dependent electrical transport measurements led to the determination of eϕ_SB_ = 135 meV at the nickel/SnS_2_ interface. Moreover, we demonstrated the extrinsic type of the SnS_2_-based phototransistor with a wide range of light response and a high photoresponsivity of approximately 0.7 mA/W.

## Methods

The bottom-gated SnS_2_ devices were fabricated on a thermally oxidized n^+^-type silicon substrate in which a 90-nm-thick SiO_2_ insulating layer offered electrical isolation from the back-gate, as well as optical detectability for the ultrathin nanosheet via optical contrast. The bottom electrode that served as both the optical indicator and the gate terminal of the transistor was pre-defined onto a silicon substrate by utilizing a standard photolithography process. We used SnS_2_ and hBN bulk solids obtained from the 2D semiconductor Inc. to generate nano-flakes using a Scotch-tape mechanical exfoliation method. We employed a technique developed in our previous work called “dry transfer,” based on a polydimethylsiloxane framework, to transfer the desired hBN flake onto the pre-patterned gold bottom-gate^2,19,25^. Subsequently, we deposited a piece of SnS_2_ on top of the hBN layer using the same technique. The SnS_2_ channel conductivity was monitored via metallization of the source/drain electrodes using a thermal evaporator system under a deposition rate of 5 Å/s to form a nickel/gold metal stack. An AFM (Park Systems, XE-100) operated under noncontact mode with a Nanosensor AR5-NCH tip was employed to characterize the topographic images of the devices. Raman signals were collected via commercially available confocal Raman spectroscopy (WiTec, alpha 300) with the excitation laser line of λ = 488 nm in ambient conditions.

The electrical transport properties of the SnS_2_/hBN devices were obtained with a semiconductor parameter analyzer (Hewlett Packard, 4156 A) in a vacuum cryostat (ASK, 700 K) under a pressure of 10^−3^ Torr. The photo-induced I–V measurements were conducted similarly under ambient conditions. To probe the photocurrent measurements, light wavelength λ spectra ranging from 300 to 1000 nm were generated by a system that consisted of a 300 W Xenon Arc lamp, a power supply (Newport, 69911), and an automated 1/8 m monochromator (Newport, 74004) with double grating. The excitation light intensity was recorded through a silicon photodiode detector (Newport, 918D-UV-OD3) mounted optical power meter (Newport, 1918-C). The collected power and irradiance data as function of photon wavelength is given in Fig. S4(b). During the photoresponse characterization, the device (active area, ~10^−7^ cm^2^) was illuminated by a monochromatic light guided by fused silica fiber optic bundle (Newport, 77577) with typical 3 mm in diameter uniform beam.

## Electronic supplementary material

Supplementary Information
