# Supplementary material for: Locally Gated SnS2/hBN Thin Film Transistors with a Broadband Photoresponse
Source: Sci Rep. 2018 Jul 12;8:10585. doi: 10.1038/s41598-018-28765-4 (PMC6043505; doi:10.1038/s41598-018-28765-4)
Supplement: Supplementary file 1 — Supplementary Information [file 41598_2018_28765_MOESM1_ESM.doc]

**Supplementary Information for**

**Locally Gated SnS_2_/hBN Thin Film Transistors with a Broadband Photoresponse**

Dongil Chu,^†^ Sang Woo Pak and Eun Kyu Kim^*^

*Quantum-Function Research Laboratory and Department of Physics, Hanyang University,*

*Seoul 04763, South Korea*

† Previously known as Dongri Qiu

*Correspondence should be addressed to: [ek-kim@hanyang.ac.kr](mailto:ek-kim@hanyang.ac.kr)

**Fig. S1.** Raman signal acquired from the as-exfoliated SnS_2_, the weak peak corresponding to in-plane vibration E_g_ located at 213.5 cm-1.

**Fig. S2.** (a) Semi-log scale of the transfer characteristics for SnS_2_ channel device under the drain bias voltage ranges from 0.1 to 0.5 V at room temperature. (b) The hysteresis behavior of transfer curves with V_G_ sweeps from -7 to 7 V (positive sweep) and 7 to -7 V (negative sweep).

**Fig. S3.** Temperature-dependent electrical transport properties of the device. (a) Color mapping of the temperature-dependent transfer curves at V_DS_=0.1 V. (b) Arrhenius plot of the conductance with different gate bias.

**Fig. S4.** (a) Dependence of responsivity on gate voltage under 500-nm-wavelength light illumination. The power of light and V_DS_ set at 23.5 μW and 0.1 V, respectively. (b) Power (left axis, orange) and irradiance (right axis, blue) of monochromatic lights as function of photon wavelength.
